# Circ_0000658 knockdown inhibits epithelial-mesenchymal transition in bladder cancer via miR-498-induced HMGA2 downregulation

**DOI:** 10.1186/s13046-021-02175-3

**Published:** 2022-01-14

**Authors:** Feng Qiu, Qiuchen Liu, Yanfu Xia, Hengxi Jin, Yuxin Lin, Xiaojun Zhao

**Affiliations:** Department of Urology, The First Affiliated Hospital of Soochow University, No. 899, Pinghai Road, Suzhou, 215000 Jiangsu Province China

**Keywords:** Bladder cancer, Epithelial-mesenchymal transition, Circular RNA_0000658, microRNA-498, High mobility group AT-hook 2, Oncogenic phenotype, In vitro and in vivo

## Abstract

**Background:**

Epithelial-mesenchymal transition (EMT) has been associated with the angiogenesis and oncogenic phenotypes of multiple malignant tumors including bladder cancer (BCa). Circular RNAs (circRNAs) are recognized as crucial regulators in the EMT. This study aims to illustrate the possible role of circular RNA_0000658 (circ_0000658) in BCa and the underlying molecular mechanism.

**Methods:**

The expression of circ_0000658, microRNA (miR)-498, and high mobility group AT-hook 2 (HMGA2) was assessed in cancer and adjacent normal tissue collected from BCa patients and human BCa cell lines (MGH-U3, T24, 5637 and SW780). BCa cells were transduced with a series of overexpression or shRNA plasmids to clarify the function of circ_0000658 and miR-498 on the oncogenic phenotypes and EMT of BCa cells. Further, we established nude mice xenografted with BCa cells to validate the roles of circ_0000658 on tumor growth in vivo.

**Results:**

Circ_0000658 was highly expressed in BCa tissue samples and cell lines, which indicated a poor prognosis of BCa patients. Circ_0000658 competitively bound to miR-498 and thus restricted miR-498 expression. Meanwhile, circ_0000658 weakened the binding of miR-498 to the target gene HMGA2 and upregulated the HMGA2 expression. Circ_0000658 elevation or miR-498 knockdown augmented oncogenic phenotypes and EMT of BCa cells, corresponding to a reduction in the expression of β-catenin and E-cadherin as well as an increase in the expression of N-cadherin, Slug, Snail, ZEB1 and Twist. Inhibition of HMGA2 reversed the effects of circ_0000658 overexpression on tumor growth in vivo.

**Conclusion:**

Altogether, our study uncovered the tumor-promoting role of circ_0000658 in BCa via the miR-498/HMGA2 axis.

**Supplementary Information:**

The online version contains supplementary material available at 10.1186/s13046-021-02175-3.

## Background

Bladder cancer (BCa) is the 10th most frequent cancer across the globe with multiple histological types, mainly including urothelial cancer and non-urothelial BCa, associated with high fatality [1, 2]. BCa develops on the urogenital tract (papillary or nonpapillary), which correspond to clinically different kinds of the disease [3]. The biological process from reduction of epithelial cell identity to acquirement of mesenchymal phenotyping is regarded as epithelial-mesenchymal transition (EMT) [4]. The initiation of EMT can facilitate the angiogenesis and oncogenic phenotype of multiple malignant tumors, including BCa [5]. Thus, finding possible molecular targets for inhibiting EMT would be of high clinical significance for the understanding of BCa pathogenesis.

Circular RNAs (circRNAs) are a class of non-coding RNAs formed by covalently closed loops through back-splicing and exon-skipping, which confer pivotal effects on plenty of biological functions, acting as microRNA (miR) sponges and reservoirs, as well as combining with RNA-binding proteins during cancer progression, including BCa [6]. For example, circEHBP1 elevation occurs in BCa with positive correlation with lymphatic metastasis and dismal prognosis of patients with BCa [6]. Ectopic expression of circRNA-MYLK accelerates the growth dynamics and EMT of BCa cells [7]. In silico analysis in our work revealed the overexpression of circ_0000658 in BCa, though the role of circ_0000658 was rarely investigated. CircRNAs could exert function on by binding to miRNAs; for example, circGLIS3 was demonstrated to augment BCa cell proliferative capacity by binding to miR-1273f [8]. miR-498 was identified to be bound to by circ_0000658 based on our bioinformatic analysis. Although rarely studied in BCa, the tumor-suppressing roles of miR-498 have been revealed in other cancers [9, 10].

Furthermore, miR-498 has been proposed to target the 3′-untranslated region (UTR) of high mobility group AT-hook 2 (HMGA2) and inhibits its translation in non-small cell lung cancer [11]. As a non-histone architectural transcription factor, HMGA2 regulates gene transcription by connecting AT-rich sequences in minor groove of B-form DNA and changes the chromatin structure [12]. Upregulated HMGA2 predicts dismal overall survival for BCa patients, serving as biomarkers for cisplatin resistance [13]. Based on the references, we posed a speculation that circ_0000658 may modulate the EMT of BCa cells via the miR-498/HMGA2 axis. Therefore, we performed loss- and gain- function assays in BCa cells and nude mouse xenografted with BCa cells to validate the possible speculation.

## Material and methods

### Ethical statement

The current study was approved by the Ethics Committee of The First Affiliated Hospital of Soochow University and conducted in strict accordance with the *Declaration of Helsinki*. All participants or their families signed informed consent documentation before sample collection. Animal experiments were performed under the approval of Animal Ethics Committee of The First Affiliated Hospital of Soochow University in accordance with *the Guide for the Care and Use of Laboratory animals* published by the US National Institutes of Health.

### Bioinformatics analysis

The BCa-related circRNA expression dataset GSE92675 retrieved from the Gene Expression Omnibus (GEO) database, with the platform annotation file of GPL19978, including 4 normal samples and 4 cancer tissue samples. Differential analysis was performed using R language “limma” package with |logFC| > 1 and *p* < 0.05 as the screening criterion. Meanwhile, the circBase database file was downloaded to convert the differentially expressed circRNA name to circRNA ID, and a heatmap of the top ten differentially expressed genes was drawn using R language “pheatmap” package. BCa-related circRNA was searched through circFunBase database, and intersection of differentially expressed circRNA and BCa-related circRNA was retrieved using the jvenn tool.

The downstream miRNAs of circRNA were predicted through RegRNA 2.0 (score ≥ 120& free_energy ≤ − 20) and circInteractome, and then intersected miRNA served as a candidate miRNA. The downstream target genes of miRNA were using bioinformatics tools StarBase and miRDB (Score > 70). BCa-related genes were analyzed using GeneCards database (Score ≥ 25) and intersected with the downstream target genes of miRNAs to identify the candidate gene. The expression of candidate genes was obtained in BCa and normal samples through the StarBase website.

### Clinical sample collection

Cancer tissues and adjacent normal tissues were surgically collected from 50 patients with BCa (Table S1) at The First Affiliated Hospital of Soochow University from January 2015 to January 2017, including 39 males and 11 females, aged between 42 to 79 years old, with an average age of 62.54 ± 11.20 years old. Four pairs of frozen samples of BCa tissues and adjacent normal tissues were selected for circRNA analysis. The samples were postoperatively pathologically confirmed. None of the patients had anti-tumor therapy prior to operation. All patients were followed up for 36 months from post-operation to January 2020. Clinical pathology referred to World Health Organization/International Society of Urological Pathology (WHO/ISUP) [14] and International Union Against Cancer (UICC) tumor node metastasis (TNM) classification.

### Immunohistochemistry

Clinical tissue specimens were collected for laser microdissection to obtain purer tumor tissues. After 3% methanol H_2_O_2_ treatment, antigen was retrieved from fixed and paraffin-embedded tissue sections. Following blocking in normal goat serum, specimens were immunostained with primary rabbit anti-human HMGA2 (ab207301, 1: 1000; Abcam) overnight at 4 °C. Incubation was further carried out with horseradish peroxidase-labeled secondary antibody goat anti-rabbit immunoglobulin G (IgG; ab6785, 1:1000, Abcam). After exposure to DAB, specimens were counterstained with hematoxylin. Observation and photographing were carried out by a microscopy with randomly selected 5 high-powered fields of view in each section and 100 cells at each field.

### RT-qPCR

Total RNA was isolated from tissues and cells using TRIzol reagent (9108Q, Takara, Japan), followed by reverse transcription into cDNA using a miRNA First-Strand cDNA Synthesis Kit (Tailing Reaction) (B532451, Sangon Biotechnology, Shanghai, China) and MightyScript First-Strand cDNA Synthesis Master Mix Kit (B639251, Sangon). The RNA was diluted to 10 folds, and 2 μL of cDNA products was collected as a template for PCR amplification, which was conducted using AceQ® qPCR SYBR Green Master Mix PCR (Q111, Vazyme, China). The gene quantification was normalized to GAPDH or U6 (Table S2) using a 2^-ΔΔCt^ method.

### Cell culture and transfection

Normal bladder epithelial cell line (SV-HUC-1) and BCa cell lines (MGH-U3, T24, 5637, SW780) were selected for this study. MGH-U3 cell line was purchased from CoBioer Biosciences CO., LTD (Nanjing, China), while the remaining cell lines were purchased from American Type Culture Collection (Manassas, VA). Culture of cells was carried out in RPMI-1640 (Gibco, Carlsbad, CA) containing 10% FBS, 10 μg/mL streptomycin and 100 U/mL penicillin at 37 °C and 5% CO_2_.

Cells in the logarithmic phase were trypsinized, seeded in a 6-well plate at a density of 1 × 10^5^ cells/well and cultured for 24 h. At 75% confluence, cells were transduced with short hairpin RNA (sh)-negative control (NC), sh-circ_0000658, overexpression (oe)-NC, oe-circ_0000658 by Lipofectamine 2000 reagent (Invitrogen, Carlsbad, CA). Next, 48 h after transfection, the transfection efficiency of sh-circ_0000658 was validated by RT-qPCR. The expression plasmid was purchased from GenePharma (Shanghai, China) at the concentration of 50 ng/mL. The core plasmid (PLKO.1) of silencing sequence of the inserted target gene and the core plasmid (pHAGE-CMV-MCS-IzsGreen) of the cDNA sequence of the inserted target gene were purchased from GenePharma (Shanghai, China), and the plasmid concentration used was 50 ng/mL.

### Western blot analysis

Cell lysis was carried out in enhanced RIPA lysis buffer containing protease inhibitors. Next, the protein concentration was quantified with a BCA Kit (BOSTER, China). The isolated protein by 10% sodium dodecyl sulfate polyacrylamide gel electrophoresis was electro-transferred onto polyvinylidene fluoride membrane. The membrane was blocked with 5% BSA and probed overnight at 4 °C with diluted primary antibodies against HMGA2 (rabbit, ab97276, 1:1000, Abcam), E-cadherin (rabbit, ab40772, 1:10000, Abcam), β-catenin (rabbit, ab32572, 1:5000, Abcam), Slug (rabbit, ab27568, 1:1000, Abcam), Snail (rabbit, ab216347, 1:1000, Abcam), ZEB1 (ab203829, 1:500, Abcam), Twist (mouse, ab50581, ab175430, 1:1000, Abcam), N-cadherin (rabbit, ab76011, 1:5000, Abcam), β-actin (rabbit, ab8227, 1:5000, Abcam). The following day, the membrane was re-probed with HRP-labeled goat anti-mouse secondary antibody (ab205719; 1: 2000; Abcam) or goat anti-rabbit (ab205718, 1:2000, Abcam) at room temperature for 1 h. The immunocomplexes on the membrane were visualized using ECL reagent (EMD Millipore, Bedford, Massachusetts) and band intensities were quantified using Image J software, with β-actin as a loading control.

### EdU assay

The cells to be tested were seeded in a 24-well plate. The culture medium with EdU at a concentration of 10 μmol/L was applied to incubate cells for 2 h. Following incubation with 100 μL of staining solution for 30 min, cells were subjected to DAPI development to stain the nucleus, which were observed by a fluorescence microscope .

### Scratch test

At intervals of 0.5–1 cm on the bottom of the 6-well plate, horizontal lines were created, with at least five lines through each well. Cells were seeded to the 6-well plate at a density of 5 × 10^5^ cells/well. The sterile 10 μL pipette tip was perpendicular to the horizontal line to the scratch on the back. The scratched cells were photographed with an inverted microscope at 0, 6, 12, and 24 h to observe migration of cells.

### Transwell assay

The apical chamber surface of the bottom membrane was coated with Matrigel, which was polymerized into a gel. Culture medium containing 10% FBS was inserted in the basolateral chamber. Next, 100 μL of cell suspension was incubated in the chambers at 37 °C for 24 h, and cells failing to invade the surface of the Matrigel membrane were discarded. Fixed cells with 4% paraformaldehyde were stained with 1% crystal violet, which were observed and counted by an inverted light microscope using ImageJ software.

### Fluorescence in situ hybridization (FISH) assay

The FITC-circ_0000658 and Cy3-miR-498 probes from Ribobio (Guangzhou, China) were used to analyze the localization in the cells or tissues. Normal bladder epithelial cells (1 × 10^5^ cells/well) were seeded onto a 6-well culture plate and cultured for 1 day. Upon reaching 80% cell confluence, the cells were fixed with 4% paraformaldehyde. Sections were subjected to 250 μL of pre-hybridization solution at 42 °C for 1 h, and then to 250 μL hybridization solution containing probe (300 ng/mL) overnight at 42 °C. Nucleus was stained with PBST-diluted DAPI (1: 800). Finally, the sections were mounted with anti-fluorescence quencher, and observed under a fluorescence microscope.

### RNA binding protein immunoprecipitation (RIP)

The binding of miR-498 and HMGA2 protein was determined using RIP kit (Millipore, Billerica, MA). The lysed cells were centrifuged to obtain the supernatant. Next, 10 μL of cell extract was taken out as input, and the other portion of the cell extract was incubated with antibody for co-precipitation. The sample and Input were digested with proteinase K, and RNA was extracted for subsequent RT-qPCR detection of HMGA2. The antibodies used were: rabbit anti-HMGA2 (1: 100, ab97276, Abcam) and rabbit anti-human IgG (1:100, ab109489, Abcam; NC).

### RNA pull-down assay

BCa cells were transfected with wild type (WT) and mutant (MUT) biotinylated circ_0000658 (50 nM each). After 48 h of transfection, the cells were collected, vortexed, and incubated with cell lysis buffer (Ambion, Austin, Texas) for 10 min. Next, 50 mL of sample cell lysate was aliquoted. The remaining lysate was incubated with M-280 streptavidin magnetic beads (Sigma) pre-coated with RNase-free and yeast tRNA (Sigma) for 3 h at 4 °C, then wash twice with cold lysis buffer, three times with low-salt buffer, and once with high-salt buffer. Finally, the total protein was extracted.

### Dual-luciferase reporter gene assay

The predicted fragments and MUT fragments of circ_0000658 or HMGA2 with miR-498 binding sites were inserted into the luciferase reporter vector as reporter plasmids circ_0000658-WT, circ_0000658-MUT, HMGA2-WT and HMGA2-MUT, respectively. The reporter plasmids were then co-transfected with mimic NC or miR-498 mimic into 293 T cells (Oulu Biotecnology, Guangzhou, China) to analyze whether miR-498 can bind to circ_0000658 or HMGA2. Following 48-h transfection, the cells were lysed and subjected to the luciferase detection kit (K801–200, Biovision, Milpitas CA), with luciferase activity normalized to renilla luciferase activity.

### Nude mouse xenografted with BCa cells

A total of 18 healthy nude mice (Beijing Institute of Pharmacology, Chinese Academy of Sciences, Beijing, China) aged 6–8 weeks old were raised in a Specific Pathogen Free animal laboratory with humidity of 60–65% at 22–25 °C. The animals were provided with free access to food and water under a 12-h light and dark cycle. After one-week acclimatization, nude mice were subcutaneously injected with T24 cells transduced with plasmids containing oe-NC + sh-NC, oe-circ_0000658 + sh-NC or oe-circ_0000658 + sh-HMGA2 (1 × 10^9^ pfu/100 μL) (*n* = 6/group). After 6 weeks, the nude mice were euthanized, with tumor volume and weight recorded. The expression of HMGA2 and tumor metastasis markers was detremined.

### Statistical analysis

SPSS 21.0 software (IBM Corp., Armonk, NY) was used for data processing. Measurement data were presented as mean ± standard deviation. Paired or unpaired t-tests were applied for the data comparison between two groups. One-way analysis of variance (ANOVA) with Tukey’s post hoc tests was used to compare data among multiple groups. Bonferroni-corrected repeated measures ANOVA was used for data comparison at different time points. Kaplan-Meier analysis was used to calculate the survival rate of patients, and the survival rates were compared with Log-rank test. The correlation of indicators was observed using Pearson correlation analysis. In all statistical analysis, a value of *p* < 0.05 represents statistical significance.

## Results

### Circ_0000658/miR-498/HMGA2 axis is predicted to play a role in the progression of BCa

**Fig. 1:**
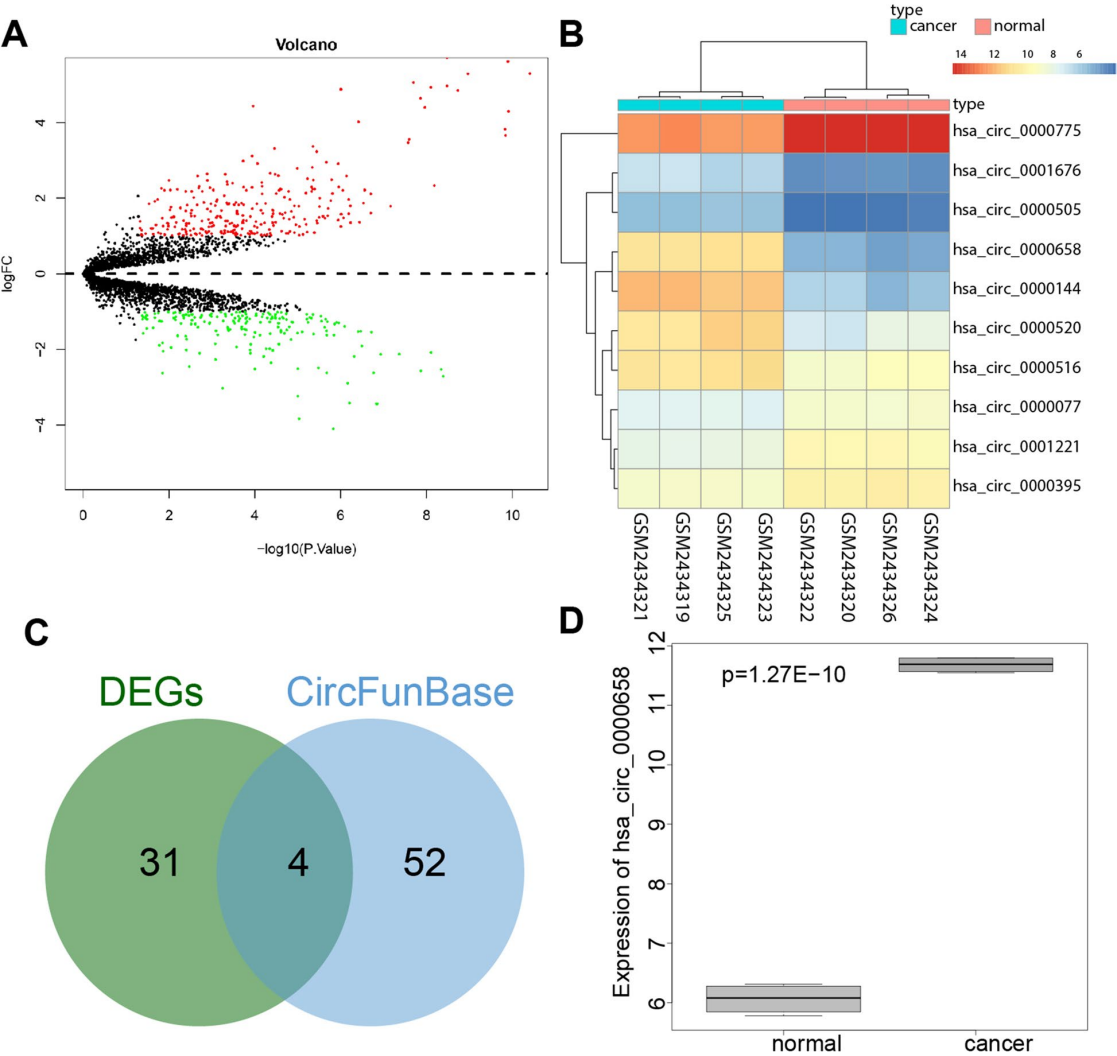
CircRNA expression profiles in BCa. **A** The volcano map of differentially expressed genes in the circRNA expression BCa-related dataset GSE92675 (red dots indicate differentially upregulated genes, green dots indicate differentially downregulated genes, x-axis indicates -log10 (*P* Value, *n* = 4), while y-axis indicates logFC); **B** A heatmap of the expression of the top 10 significantly differentially expressed circRNAs; **C** Venn diagram of differentially expressed circRNAs and BCa-related circRNAs; **D** A box diagram of the expression of hsa_circ_0000658 in BCa compared to normal samples in GSE92675 dataset (*n* = 4)

The differential analysis on the circRNA expression dataset GSE92675 related to BCa yielded 461 differentially expressed genes (Fig. 1A). After circRNA ID conversion, 35 differentially expressed circRNAs were obtained and the expression heatmap was drawn based on the top 10 circRNA (Fig. 1B). Next, 56 BCa-related circRNAs were obtained through the circFunBase database, and intersected with differentially expressed circRNAs, after which, we obtained four candidate circRNAs: hsa_circ_0000658, hsa_circ_0000144, hsa_circ_0000520, and hsa_circ_0001336 (Fig. 1C). Among them, the expression of hsa_circ_0000658 was the most significantly highly expressed in BCa as reflected by the dataset GSE92675 (Fig. 1D). Therefore, circ_0000658 was speculated to be highly expressed in BCa and adopted for further study.

### Circ_0000658 is highly expressed in BCa tissues and cells and this high expression indicates poor prognosis of BCa patients

**Fig. 2:**
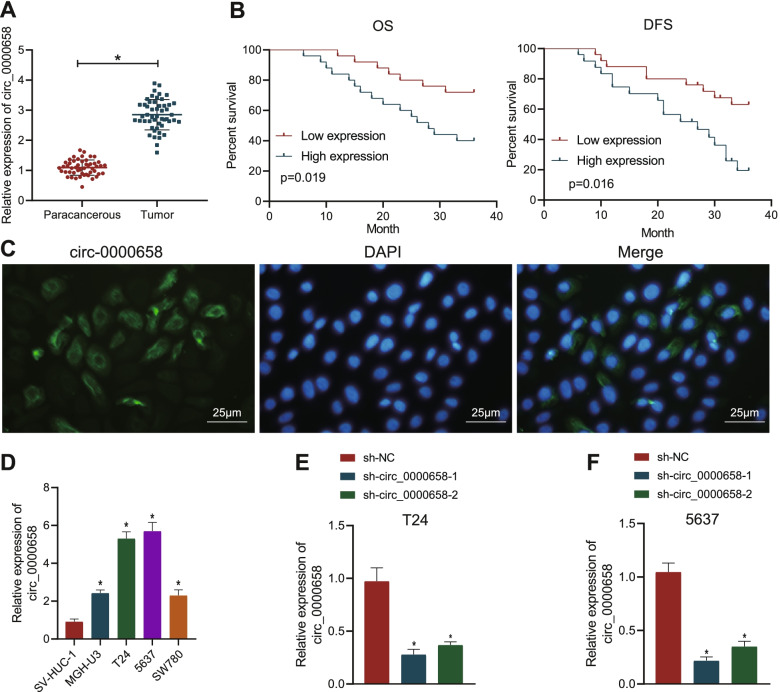
Circ_0000658 is overexpressed in BCa tissues and cells and this overexpression is associated with poor prognosis of BCa patients. **A** The expression of circ_0000658 in BCa tissues and adjacent normal tissues determined with RT-qPCR (*n* = 50, *p < 0.001 vs. adjacent normal tissues); **B** The correlation between the expression of circ_0000658 and DFS and OS of patients with BCa analyzed with Kaplan-Meier analysis; **C** The location of circ_0000658 in cells determined with FISH assay; **D** The expression of circ_0000658 in BCa cells MGH-U3, T24, 5637, SW780 and normal bladder epithelial cell line SV-HUC-1 assessed with RT-qPCR (*p < 0.05 vs. SV-HUC-1 cells); **E** Silencing efficiency of circ_0000658 in T24 cells validated using RT-qPCR; **F** Silencing efficiency of circ_0000658 in 5637 cells validated using RT-qPCR (*p < 0.05 vs. sh-NC). All cell experiments were repeated three times

We validated that expression of circ_0000658 was upregulated in the BCa tissues relative to adjacent normal tissues (Fig. 2A). As reflected by Kaplan-Meier analysis, the overall survival (OS) and progression-free survival (DFS) of patients with elevated circ_0000658 were significantly lower than those with downregulated circ_0000658, suggesting that elevation of circ_0000658 was related to dismal prognosis of BCa patients (Fig. 2B).

FISH assay further demonstrated that circ_0000658 was mainly expressed in the cytoplasm (Fig. 2C). It was found that circ_0000658 elevation occurred in BCa cell lines compared with SV-HUC-1 cells. More significantly upregulated circ_0000658 was observed in T24 and 5637 cells, which were selected for subsequent experiments (Fig. 2D). We then silenced circ_0000658 expression and RT-qPCR successfully validated its silencing efficiency. sh-circ_0000658–1 was selected for subsequent use due to better silencing efficiency (Fig. 2E, F). Together, circ_0000658 is highly expressed in BCa tissues and cells, which is related to the poor prognosis of BCa patients.

### Circ_0000658 promotes growth dynamics of BCa cells

**Fig. 3:**
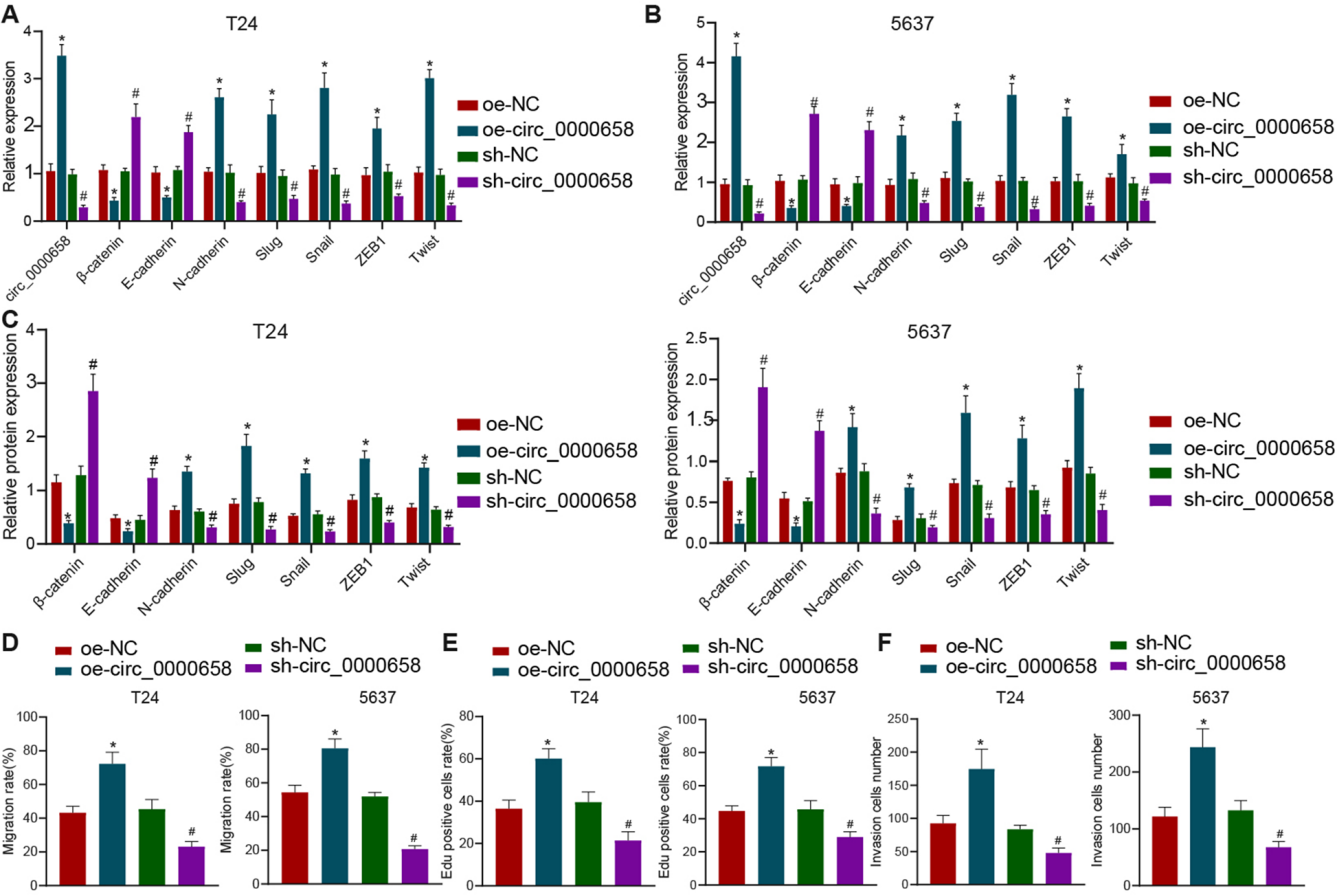
Circ_0000658 promotes the oncogenic phenotypes of BCa cells. **A** The expression of circ_0000658 and EMT markers β-catenin, E-cadherin, N-cadherin, Slug, Snail, ZEB1 and Twist in T24 cells determined with RT-qPCR; **B** The expression of circ_0000658 and EMT markers β-catenin, E-cadherin, N-cadherin, Slug, Snail, ZEB1 and Twist in 5637 cells determined with RT-qPCR; **C** The expression of circ_0000658 and EMT markers β-catenin, E-cadherin, N-cadherin, Slug, Snail, ZEB1 and Twist in T24 and 5637 cells determined with Western blot analysis; **D** The cell migration ability of T24 and 5637 cells measured with scratch test. **E** The cell proliferation of T24 and 5637 cells after circ_0000658 silencing determined with EdU assay; **F** The cell invasion of T24 and 5637 cells determined with Transwell assay. *p < 0.05 vs. oe-NC; #*p* < 0.05 vs. sh-NC. All cell experiments were repeated three times

To define the role circ_0000658 in the progression of BCa, we overexpressed or knocked down circ_0000658 in T24 and 5637 cells, respectively. RT-qPCR successfully verified the efficiency of oe-circ_0000658. Further, overexpressed circ_0000658 reduced the expression of β-catenin and E-cadherin, while increasing that of N-cadherin, Slug, Snail, ZEB1 and Twist, which were negated in response to sh-circ_0000658 in T24 and 5637 cells (Fig. 3A-C, Fig. S1A), suggesting that circ_0000658 could augment the EMT of BCa cells.

Moreover, the results of scratch test, EdU and Transwell assay revealed that forced circ_0000658 expression augmented the migratory, proliferative, and invasive capacities of BCa cells while silencing of circ_0000658 caused opposite results (Fig. 3D-F, Fig. S1B-D). In summary, circ_0000658 accelerated EMT and oncogenic phenotypes of BCa cells, thus playing a promising role in the growth of BCa.

### Circ_0000658 competitively binds to miR-498 and inhibits its expression

**Fig. 4:**
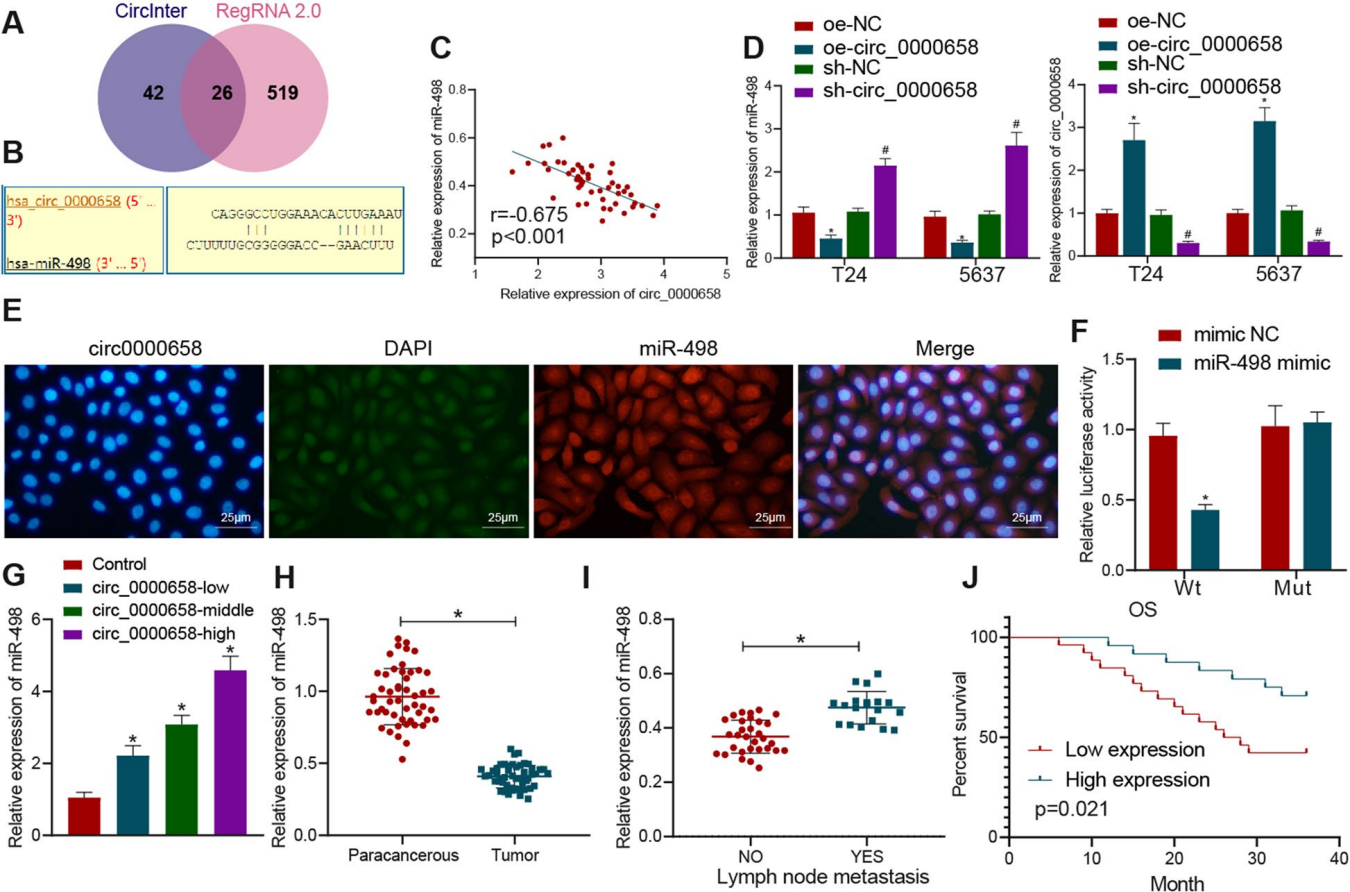
Circ_0000658 competitively binds to miR-498 and reduces its expression. **A** Venn diagram of the intersection of downstream miRNAs targeted by circ_0000658 predicted using RegRNA 2.0 and circInteractome; **B** The binding site of circ_0000658 and miR-498 predicted using circInteractome; **C** Correlation analysis of circ_0000658 and miR-498 expression in BCa tissues; **D** miR-498 and circ_0000658 expression in T24 and 5637 cell lines determined with RT-qPCR (*p < 0.05 vs. oe-NC; #*p* < 0.05 vs. sh-NC); **E** Subcellular localization of circ_0000658 and miR-498 assayed with FISH; **F** Targeting relationship between circ_0000658 and miR-498 verified using dual-luciferase reporter gene assay (right panel) (*p < 0.05 vs. mimic NC); **G** The enrichment of circ_0000658 to miR-498 determined with RNA pull-down (*p < 0.05 vs. control); **H** The expression of miR-498 in BCa tissues and adjacent normal tissues (*p < 0.05 vs. adjacent normal tissues); **I** miR-498 expression in BCa tissues with or without lymphatic metastasis determined with RT-qPCR (*p < 0.05 vs. BCa tissues without lymphatic metastasis); **J** The relationship between BCa patients’ prognosis and miR-498 expression analyzed by the Kaplan-Meier method (*p < 0.05 vs. low miR-498 expression group). All cell experiments were repeated three times

Subsequently, the downstream miRNAs of circ_0000658 were predicted by RegRNA 2.0 and circInteractome, and the overlapping results indicated 26 candidate miRNAs (Fig. 4A, Table S3). Of note, the binding sites of circ_0000658 and miR-498 had a higher context ^+^ score and miR-498 has been rarely studied in the BCa. We then obtained the binding sites between circ_0000658 and miR-498 through circInteractome (Fig. 4B). Further, expression of circ_0000658 and miR-498 was negatively correlated (Fig. 4C).

Additionally, RT-qPCR analysis suggested that miR-498 was diminished in response to oe-circ_0000658, while an opposite effect observed in response to sh-circ_0000658 (Fig. 4D). As reflected by FISH assay, circ_0000658 and miR-498 were co-localized in the cytoplasm (Fig. 4E). Luciferase activity assay suggested that forced miR-498 expression reduced the luciferase activity of circ_0000658-WT without altering that of circ_0000658-MUT (Fig. 4F). Moreover, the results of RIP and RNA- pull-down revealed that circ_0000658 with higher concentration pulled down more miR-498 (Fig. 4G). The above results demonstrated a direct binding between circ_0000658 and miR-498.

RT-qPCR data demonstrated lower level of miR-498 in BCa tissues than that in adjacent normal tissues (Fig. 4H). In addition, miR-498 level in BCa with lymphatic metastasis was higher than that in BCa tissues without lymphatic metastasis, which was opposite to the general result of poor expression of miR-498 in tumors (Fig. 4I). Moreover, patients with downregulated miR-498 expression had larger tumors, shortened OS accompanied by lymph node metastasis relative to patients with high expression of miR-498 (Fig. 4J). Coherently, circ_0000658 could adversely regulate miR-498 expression, and downregulated miR-498 is significantly related to dismal prognosis of BCa patients.

### miR-498 targets HMGA2

**Fig. 5:**
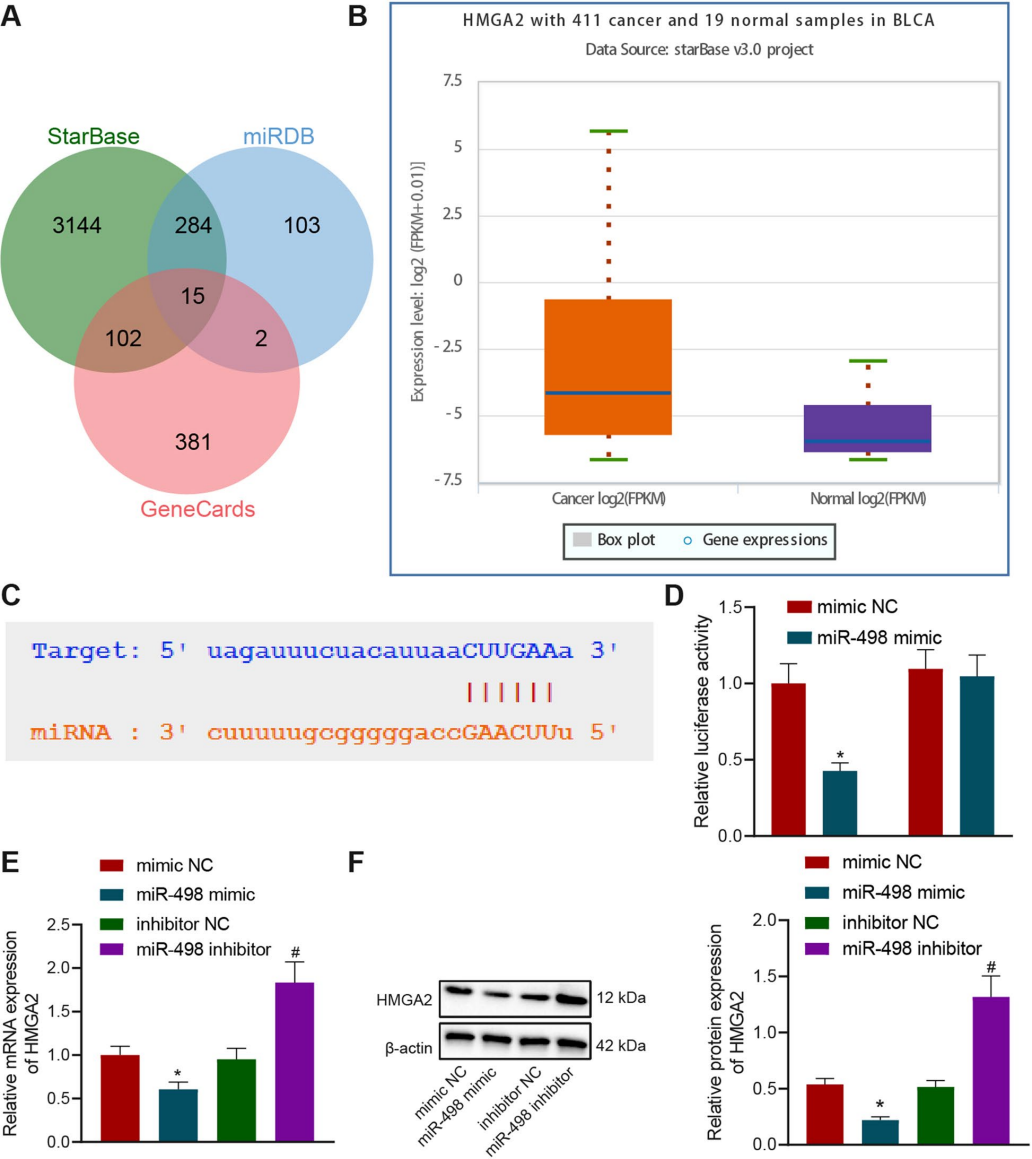
miR-498 targets HMGA2 and negatively regulates HMGA2 expression. **A** Venn diagram of the downstream target genes of miR-498 predicted by StarBase and miRDB predict and BCa-related genes obtained from the GeneCards database; **B** A box diagram of HMGA2 expression in BCa compared with normal samples analyzed by StarBase website; **C** Putative miR-498 binding sites in the 3′-UTR of HMGA2 mRNA predicted by the StarBase website; **D** The targeting relationship between miR-498 and HMGA2 determined with dual-luciferase reporter gene assay. **E** The expression of HMGA2 in BCa cells determined with RT-qPCR; **F** Representative Western blots of HMGA2 protein and its quantitation in the BCa cells. *p < 0.05 vs. mimic NC. # *p* < 0.05 vs. inhibitor NC. All cell experiments were repeated three times

The downstream target genes of miR-498 were predicted by means of StarBase and miRDB and intersected with the BCa-related genes in the GeneCards database, which yielded 15 candidate target genes (Fig. 5A, Table S4). Among them, HMGA2 expression has been indicated to be overexpressed in BCa and involved in the EMT process [14, 15]. We also obtained the upregulation of HMGA2 in BCa samples versuse normal samples through the StarBase website (Fig. 5B), and identified binding sites between miR-498 and HMGA2 (Fig. 5C). We further confirmed that forced miR-498 expression restricted the luciferase activity of HMGA2-WT without altering that of HMGA2-MUT (Fig. 5D). Subsequently, we indicated that HMGA2 was diminished in response to miR-498 mimic, while opposite trends were observed in response to miR-498 inhibitor (Fig. 5E, F). Collectively, miR-498 can target HMGA2 and reduce its expression.

### Circ_0000658 upregulates HMGA2 by downregulating miR-498 to augment malignant phenotypes of BCa cells

**Fig. 6:**
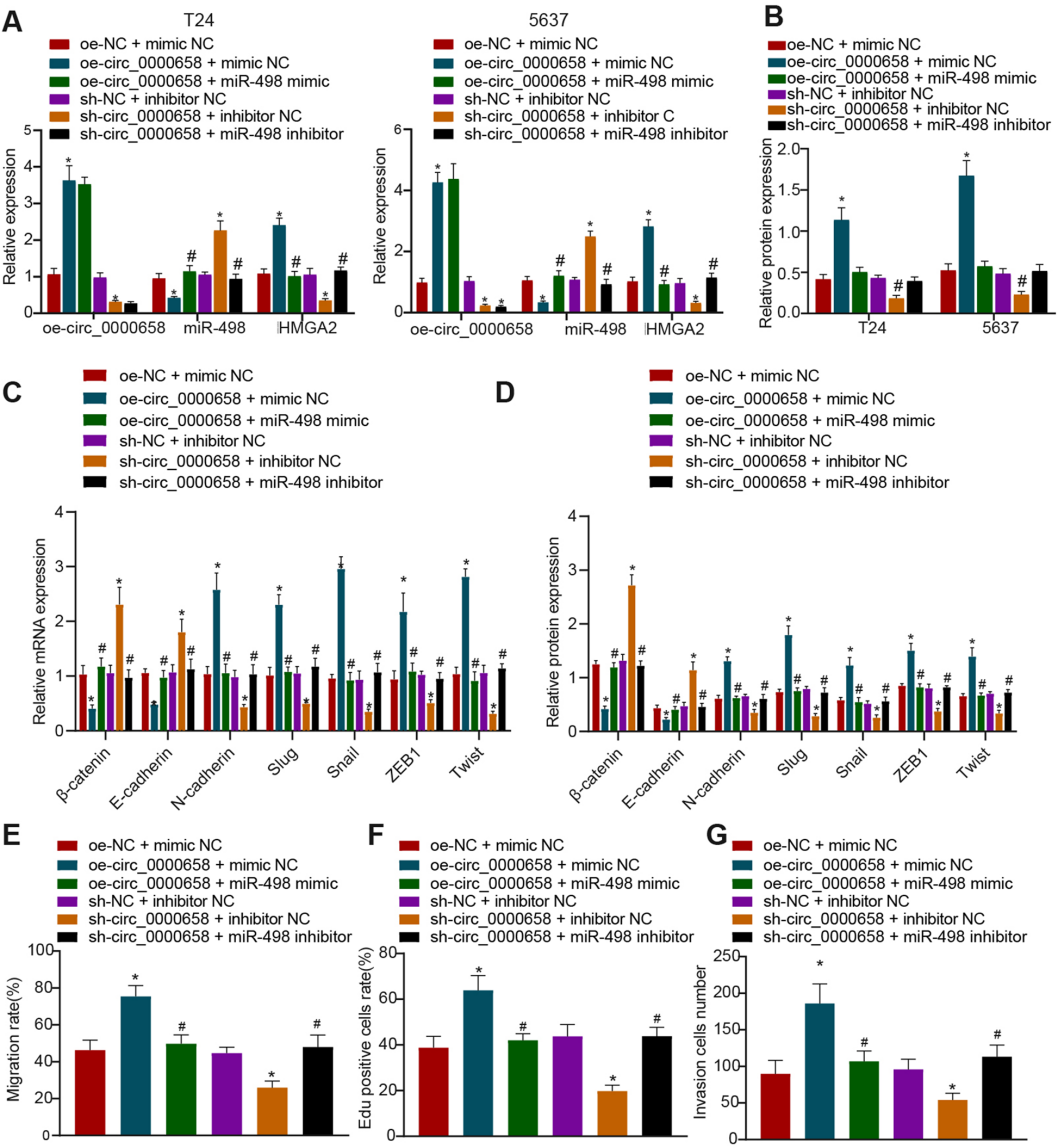
Circ_0000658 upregulates HMGA2 by downregulating miR-498 to augment EMT, proliferation, invasion and migration of BCa cells. Circ_0000658 was overexpressed or silenced in the presence or absence of miR-498 mimic or miR-498 inhibitor in T24 and 5637 cells. **A** The expression of circ_0000658, miR-498, and HMGA2 in T24 and 5637 cells determined with RT-qPCR; **B** Western blot analysis of protein expression of HMGA2 in T24 and 5637 cells; **C** The mRNA expression of EMT markers β-catenin, E-cadherin, N-cadherin, Slug, Snail, ZEB1 and Twist in T24 and 5637 cells determined with RT-qPCR; **D** The protein expression of EMT markers β-catenin, E-cadherin, N-cadherin, Slug, Snail, ZEB1 and Twist in T24 and 5637 cells determined with Western blot analysis; **E** The cell migration ability of T24 and 5637 cells measured with scratch test; **F** The cell proliferation of T24 and 5637 cells after circ_0000658 silencing determined with EdU assay; **G** The cell invasion of T24 and 5637 cells determined with Transwell assay. *p < 0.05 vs. oe-NC + mimic NC or sh-NC + inhibitor NC; #*p* < 0.05 vs. oe-circ_0000658 + mimic NC or sh-circ_0000658 + inhibitor NC. All cell experiments were repeated three times

To further clarify the regulatory role of circ_0000658 in miR-498/HMGA2 axis, we overexpressed or silenced circ_0000658 expression in the presence or absence of miR-498 mimic or miR-498 inhibitor in T24 and 5637 cells. It was demonstrated that oe-circ_0000658 upregulated circ_0000658 and HMGA2 while reducing miR-498. In contrast, further miR-498 overexpression diminished HMGA2 expression. The sh-circ_0000658 triggered a decline in the circ_0000658 and HMGA2 expression as well as an increase in the miR-498 expression, whereas HMGA2 expression was elevated upon further addition of miR-498 inhibitor (Fig. 6A, B, Fig. S2A).

In addition, forced circ_0000658 expression reduced the expression of β-catenin and E-cadherin, while increasing that of N-cadherin, Slug, Snail, ZEB1 and Twist. However, opposite trends were observed by the further treatment of miR-498-mimic. Silencing of circ_0000658 induced an increase in the expression of β-catenin and E-cadherin as well as a decline in that of N-cadherin, Slug, Snail, ZEB1 and Twist, while these results were reversed by the further treatment of miR-498 inhibitor (Fig. 6C, D, Fig. S2B). Moreover, forced circ_0000658 expression augmented the migratory, proliferative, and invasive capacities of BCa cells, which was abrogated by the further treatment with miR-498 mimic. Silencing of circ_0000658, however, restricted the growth dynamics of BCa cells, while the further treatment of miR-498 inhibitor reversed the inhibitory effects of circ_0000658 silencing (Fig. 6E-G, Fig. S2C-E). In summary, circ_0000658 could upregulate HMGA2 by binding to miR-498 and thus augment EMT and oncogenic phenotypes of BCa cells.

### Circ_0000658 accelerates the tumorigenesis of BCa cells by downregulating miR-498 and upregulating HMGA2 expression in vivo

**Fig. 7:**
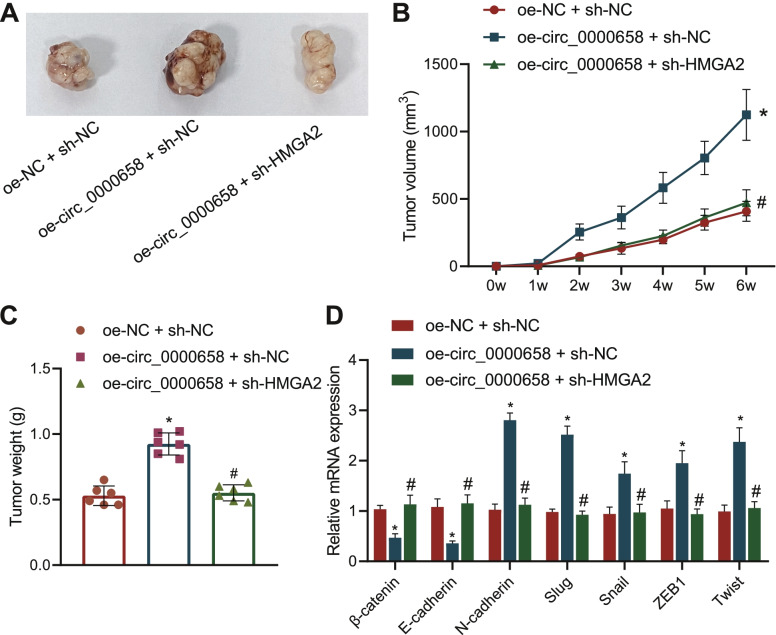
Circ_0000658 promotes the tumorigenesis of BCa cells by decreasing miR-498 expression and upregulating HMGA2 expression in vivo. **A** Representative images showing xenografts in nude mice; **B** Tumor volume growth curve of nude mice; **C** Tumor weight of nude mice; **D** The mRNA expression of EMT markers in tumor tissues of nude mice determined with RT-qPCR. *n* = 6. *p < 0.05 vs. oe-NC + sh-NC; #*p* < 0.05 vs. oe-circ_0000658 + sh-NC

To define the roles of the circ_0000658/miR-498/HMGA2 axis in vivo, we constructed a xenograft nude mouse model by subcutaneous injection of T24 cells transduced with plasmids containing oe-circ_0000658 alone or combined with sh-HMGA2 into the nude mice. Results revealed that oe-circ_0000658 + sh-NC-treated mice exhibited increased tumor volume and weight, while further inhibition of HMGA2 reversed this effect relative to oe-circ_0000658 alone (Fig. 7A-C). In addition, the tumor tissues of oe-circ_0000658-treated mice exhibited reduced β-catenin and E-cadherin, as well as elevated N-cadherin, Slug, Snail, ZEB1 and Twist, whereas further inhibition of HMGA2 reversed this effect relative to oe-circ_0000658 alone (Fig. 7D, Fig. S3). Coherently, circ_0000658 promotes the tumorigenesis of BCa cells in vivo, which was related to decrease of miR-498 and upregulation of HMGA2.

## Discussion

BCa develops on the urogenital tract (papillary or nonpapillary), which correspond to clinically different kinds of the disease [3]. EMT develops from loss of cell polarity of epithelial cells, cell-cell adhesion to mesenchymal phenotype, which exacerbates cancer progression [16]. The association between EMT and BCa progression and metastasis has been previously highlighted [17]. In the present study, we identified a novel differentially expressed circ_0000658 in the BCa progression via regulation of EMT. The present study validated that circ_0000658 could attenuate miR-498 binding to HMGA2 to augment EMT, oncogenic phenotypes of BCa cells, which could exacerbate the development of BCa.

Initially, we revealed that circ_0000658 was highly expressed in BCa tissues and cells and correlated to the poor prognosis of BCa patients. In addition, circ_0000658 augmented the growth dynamics of BCa cells, which was rarely reported. Concordantly, elevation of circEHBP1 occurred in BCa and promotes the lymphatic metastasis with dismal prognosis of BCa patients [18]. EMT is known as a process of the transdifferentiation of epithelial cells into motile mesenchymal cells, which leads pathologically to fibrosis and cancer progression [19]. EMT occurs in both physiological and pathological conditions and can be triggered by a conserved set of inducing signals, downstream effectors and transcriptional regulators, contributing to functional changes in cell migratory and invasive potential [20]. E-cadherin, N-cadherin, β-catenin, Slug, Snail, Twist, and ZEB2 are EMT markers, which predict intravesical recurrence in patients with non-muscle-invasive urothelial carcinoma of the bladder [21]. Further analysis in the current study revealed that overexpression of circ_0000658 reduced the expression of β-catenin and E-cadherin, whereas elevating that of N-cadherin, Slug, Snail, ZEB1 and Twist. Conversely, a contrary result was observed in response to sh-circ_0000658. This represents the first evidence for the regulation of the EMT by circ_0000658 in BCa and may have importance in regulating the progression of BCa. Consistent with our study, knockdown of EFEMP2 in BCa cells triggered reduction in the epithelial marker E-cadherin expression, as well as increase in mesenchymal markers N-cadherin, Snail and Slug, which is associated with augmented cell proliferative, migratory and metastatic capacities [22]. Depletion of circ_100984 retards the BCa tumor growth and migratory and invasive capacities in vitro and in vivo by inhibiting expression of EMT markers [23]. Our in vivo experiments also demonstrated that circ_0000658 increased the tumor volume and weight, which promotes the tumorigenesis of BCa cells.

Moreover, we also revealed that circ_0000658 competitively bound to and restricted miR-498 expression, although it’s rarely documented. Evidence has suggested that lncRNAs can interact with miRNAs and regulate the expression of miRNAs as a competitive endogenous non-coding RNA [24]. For instance, circ_GFRA1 has been proposed to competitively bind to miR-498 and negatively regulate the expression of miR-498 in the context of hepatocellular carcinoma [25]. In addition, circ_PRMT5 can sponge miR-498 and thus inhibit the expression of miR-498 during non-small-cell lung cancer [9]. Our results displayed that miR-498 knockdown augmented the EMT, oncogenic phenotypes of BCa cells. miRNAs exert crucial roles in the initiation and progression of cancer due to their involvement in the regulation of various biological processes, including EMT, by acting as oncomiRs or as tumor suppressors via multiple molecular mechanisms [26]. For example, miR-485-5p has been recognized as a tumor suppressor, and its ectopic expression could restrict EMT and metastasis of bladder cancer cells through targeting HMGA2 [15]. Meanwhile, miR-498 expression has been found to be reduced in liver cancer patient tissues and cell lines while its upregulation suppresses liver cancer cell proliferative and invasive capacities and EMT [27]. Inhibiting miR-498 expression can induce the EMT and proliferative potentials in non-small-cell lung cancer cells [28]. Additionally, miR-498 expression is reduced in gastric cancer associated with dismal prognosis but its abundant expression induces a reduction in EMT markers to suppress the metastatic and proliferative capacity of gastric cancer cells [10]. However, the involvement of miR-498 in BCa confirmed by the current study warrants further investigation due to the lack of available literature to support it.

miR-498 has been proposed to target the 3′-UTR of HMGA2 and inhibits its translation in non-small cell lung cancer [11]. A prior study supports that miR-498 targeted and negatively regulated HMGA2. Additionally, accumulating evidence has demonstrated the regulation of circRNAs on HMGA2 whereby circRNAs can act as miRNA sponges, and thus reduce their regulatory effects on the target mRNAs [29–31]. The current study confirmed for the first time that circ_0000658 enhanced the expression of HMGA2 by competitively binding to miR-498. We also revealed that the inhibition of HMGA2 reversed the trends of circ_0000658 overexpression on tumor volume and EMT markers in vivo. Consistently, HMGA2 expression is elevated in the BCa tissues relative to non-cancerous tissues and its inhibition is correlated with the delayed BCa progression [32]. N-cadherin expression is observed to be reduced in HMGA2-knocked down cells of gastric cancer, and HMGA2 elevation would exacerbate the invasive and metastatic potential of gastric cancer with the promoting effect on EMT [33]. In the context of colorectal cancer, the depletion of HMGA2 could abrogate the promoting effects of miR-532-3p inhibitor on cell malignancy [34].

## Conclusions

**Fig. 8:**
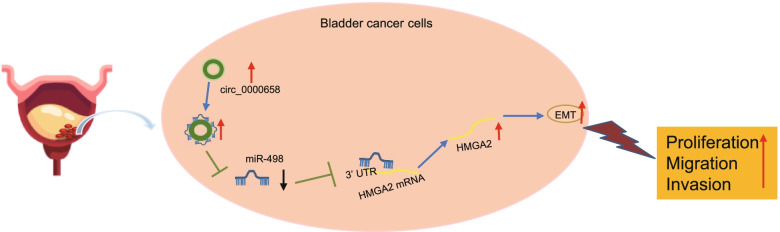
The mechanism graph of the regulatory network and function of circ_0000658 in BCa. Circ_0000658 competitively binds to miR-498 and downregulates miR-498 expression, thus upregulating HMGA2. By this mechanism, circ_0000658 stimulates EMT, proliferation, invasion and migration of BCa cells and promotes the ensuing BCa progression

In conclusion, the present study sheds new light on the mechanism underlying proliferative, migratory and invasive capacities, and EMT of BCa cells. Specifically, circ_0000658 downregulates miR-498 to augment EMT, oncogenic phenotypes of BCa cells via HMGA2 upregulation (Fig. 8). However, the major role of circ_0000658 during BCa development remains rarely elucidated due to the lack of supporting literature. Further studies are required to confirm our findings and develop clinical applications that are less vulnerable to degradation, making them novel targets for therapeutic approaches.

## Supplementary Information

**Additional file 1: Figure S1.** Representative images of Western blot analysis (A), scratch test (B), EdU assay (C) and Transwell assay (D). A: The expression of circ_0000658 and EMT markers β-catenin, E-cadherin, N-cadherin, Slug, Snail, ZEB1 and Twist in T24 and 5637 cells determined with Western blot analysis. B: The cell migration ability of T24 and 5637 cells measured with scratch test. C, The cell proliferation of T24 and 5637 cells after circ_0000658 silencing determined with EdU assay; D: The cell invasion of T24 and 5637 cells determined with Transwell assay.

**Additional file 2: Figure S2.** Representative images of Western blot assay (A-B), scratch test (C), EdU assay (D) and Transwell assay (E). A: Western blot analysis of protein expression of HMGA2 in T24 and 5637 cells. B: The protein expression of EMT markers β-catenin, E-cadherin, N-cadherin, Slug, Snail, ZEB1 and Twist in T24 and 5637 cells determined with Western blot analysis. C: The cell migration ability of T24 and 5637 cells measured with scratch test; D: The cell proliferation of T24 and 5637 cells after circ_0000658 silencing determined with EdU assay; E: The cell invasion of T24 and 5637 cells determined with Transwell assay.

**Additional file 3: Figure S3.** Determination of the protein expression of EMT markers in tumor tissues of nude mice following circ_0000658 alteration by Western blot analysis. *n* = 6. *p < 0.05 vs. oe-NC + sh-NC; #*p* < 0.05 vs. oe-circ_0000658 + sh-NC.

**Additional file 4: Table S1.** Clinicopathological characteristics of 50 patients with BCa. **Table S2.** Primer sequences used for RT-qPCR. **Table S3.** MiRNAs downstream circ_0000658 based on RegRNA 2.0 and circInteractome databases. **Table S4.** Intersection of downstream target genes of miR-498 predicted by StarBase and miRDB databases with the BCa-related genes in the GeneCards database.

## Data Availability

The data used to support the findings of this study are available from the corresponding author upon request.

## Abbreviations

BCa: Bladder cancer
circRNAs: Circular RNAs
HMGA2: High mobility group AT-hook 2
EMT: Epithelial-mesenchymal transition
miRs: MicroRNAs
3′-UTR: 3′-untranslated region
GEO: Gene Expression Omnibus
DFS: Progression-free survival
RPMI: Roswell Park Memorial Institute
FBS: Fetal bovine serum
cDNA: Complementary DNA
RT-qPCR: Reverse transcription quantitative polymerase chain reaction
miRNAs: MicroRNAs
oe: Overexpression
NC: Negative control
sh: Short hairpin RNA
DAB: 3,3′-diaminobenzidine tetrahydrochloride
WHO: World Health Organization
ISUP: International Society of Urological Pathology
UICC: International Union Against Cancer
TNM: Tumor node metastasis
PBS: Phosphate buffer saline
GAPDH: Glyceraldehyde-3-phosphate dehydrogenase
RIPA: Radio-immunoprecipitation assay
BCA: Bicinchoninic acid
IgG: Immunoglobulin G
HRP: Horseradish peroxidase
FISH: Fluorescence in situ hybridization
WT: Wild type
MUT: Mutant
ECL: Enhanced chemiluminescence
BSA: Bovine serum albumin
DAPI: 4′,6-diamidino-2-phenylindole
EdU: 5-ethynyl-2′-deoxyuridine
RIP: RNA binding protein immunoprecipitation
ANOVA: Analysis of variance

## References

[1] Afonso J, Santos LL, Longatto-Filho A, Baltazar F (2020). Competitive glucose metabolism as a target to boost bladder cancer immunotherapy. Nat Rev Urol.

[2] Alifrangis C, McGovern U, Freeman A, Powles T, Linch M (2019). Molecular and histopathology directed therapy for advanced bladder cancer. Nat Rev Urol.

[3] Czerniak B, Dinney C, McConkey D (2016). Origins of bladder cancer. Annu Rev Pathol.

[4] Georgakopoulos-Soares I, Chartoumpekis DV, Kyriazopoulou V, Zaravinos A (2020). EMT factors and metabolic pathways in cancer. Front Oncol.

[5] Niu W, Xu L, Li J, Zhai Y, Sun Z, Shi W (2020). Polyphyllin II inhibits human bladder cancer migration and invasion by regulating EMT-associated factors and MMPs. Oncol Lett.

[6] Yang L, Zou X, Zou J, Zhang G (2021). Functions of circular RNAs in bladder, prostate and renal cell cancer (review). Mol Med Rep.

[7] Zhong Z, Huang M, Lv M, He Y, Duan C, Zhang L (2017). Circular RNA MYLK as a competing endogenous RNA promotes bladder cancer progression through modulating VEGFA/VEGFR2 signaling pathway. Cancer Lett.

[8] Wu S, Yang J, Xu H, Wang X, Zhang R, Lu W, et al. Circular RNA circGLIS3 promotes bladder cancer proliferation via the miR-1273f/SKP1/Cyclin D1 axis. Cell Biol Toxicol. 2021. 10.1007/s10565-021-09591-3. 10.1007/s10565-021-09591-3 PMC8789643 33656636

[9] Wang Y, Li Y, He H, Wang F (2019). Circular RNA circ-PRMT5 facilitates non-small cell lung cancer proliferation through upregulating EZH2 via sponging miR-377/382/498. Gene.

[10] You D, Wang D, Liu P, Chu Y, Zhang X, Ding X (2020). MicroRNA-498 inhibits the proliferation, migration and invasion of gastric cancer through targeting BMI-1 and suppressing AKT pathway. Hum Cell.

[11] Gao N, Wang FX, Wang G, Zhao QS (2018). Targeting the HMGA2 oncogene by miR-498 inhibits non-small cell lung cancer biological behaviors. Eur Rev Med Pharmacol Sci.

[12] Zhang S, Mo Q, Wang X (2019). Oncological role of HMGA2 (review). Int J Oncol.

[13] Krafft U, Tschirdewahn S, Hess J, Harke NN, Hadaschik B, Olah C (2019). Validation of survivin and HMGA2 as biomarkers for cisplatin resistance in bladder cancer. Urol Oncol.

[14] Li WQ, Zhao WC, Xin J, Niu TL, Chao YF, Zhou P, et al. MicroRNA-142-3p suppresses cell proliferation and migration in bladder cancer via Rac1. J Biol Regul Homeost Agents. 2020;34. 10.23812/19-460-A. 10.23812/19-460-A 32107907

[15] Chen Z, Li Q, Wang S, Zhang J (2015). miR4855p inhibits bladder cancer metastasis by targeting HMGA2. Int J Mol Med.

[16] Lee HM, Hwang KA, Choi KC (2017). Diverse pathways of epithelial mesenchymal transition related with cancer progression and metastasis and potential effects of endocrine disrupting chemicals on epithelial mesenchymal transition process. Mol Cell Endocrinol.

[17] McConkey DJ, Choi W, Marquis L, Martin F, Williams MB, Shah J (2009). Role of epithelial-to-mesenchymal transition (EMT) in drug sensitivity and metastasis in bladder cancer. Cancer Metastasis Rev.

[18] Zhu J, Luo Y, Zhao Y, Kong Y, Zheng H, Li Y, et al. circEHBP1 promotes lymphangiogenesis and lymphatic metastasis of bladder cancer via miR-130a-3p/TGFbetaR1/VEGF-D signaling. Mol Ther. 2021. 10.1016/j.ymthe.2021.01.031. 10.1016/j.ymthe.2021.01.031 PMC8116613 33545359

[19] Lamouille S, Xu J, Derynck R (2014). Molecular mechanisms of epithelial-mesenchymal transition. Nat Rev Mol Cell Biol.

[20] Yang J, Antin P, Berx G, Blanpain C, Brabletz T, Bronner M (2020). Guidelines and definitions for research on epithelial-mesenchymal transition. Nat Rev Mol Cell Biol.

[21] Liu B, Miyake H, Nishikawa M, Fujisawa M (2015). Expression profile of epithelial-mesenchymal transition markers in non-muscle-invasive urothelial carcinoma of the bladder: correlation with intravesical recurrence following transurethral resection. Urol Oncol.

[22] Zhou Q, Chen S, Lu M, Luo Y, Wang G, Xiao Y (2019). EFEMP2 suppresses epithelial-mesenchymal transition via Wnt/beta-catenin signaling pathway in human bladder cancer. Int J Biol Sci.

[23] Tong L, Yang H, Xiong W, Tang G, Zu X, Qi L (2021). circ_100984-miR-432-3p axis regulated c-Jun/YBX-1/beta-catenin feedback loop promotes bladder cancer progression. Cancer Sci.

[24] Chen K, Ma Y, Wu S, Zhuang Y, Liu X, Lv L (2019). Construction and analysis of a lncRNAmiRNAmRNA network based on competitive endogenous RNA reveals functional lncRNAs in diabetic cardiomyopathy. Mol Med Rep.

[25] Lv S, Li Y, Ning H, Zhang M, Jia Q, Wang X (2021). CircRNA GFRA1 promotes hepatocellular carcinoma progression by modulating the miR-498/NAP1L3 axis. Sci Rep.

[26] Markopoulos GS, Roupakia E, Tokamani M, Chavdoula E, Hatziapostolou M, Polytarchou C (2017). A step-by-step microRNA guide to cancer development and metastasis. Cell Oncol (Dordr).

[27] Zhang X, Xu X, Ge G, Zang X, Shao M, Zou S (2019). miR498 inhibits the growth and metastasis of liver cancer by targeting ZEB2. Oncol Rep.

[28] Ji X, Tao R, Sun LY, Xu XL, Ling W (2020). Down-regulation of long non-coding RNA DUXAP8 suppresses proliferation, metastasis and EMT by modulating miR-498 through TRIM44-mediated AKT/mTOR pathway in non-small-cell lung cancer. Eur Rev Med Pharmacol Sci.

[29] Cai X, Nie J, Chen L, Yu F (2020). Circ_0000267 promotes gastric cancer progression via sponging MiR-503-5p and regulating HMGA2 expression. Mol Genet Genomic Med.

[30] Liu K, Mou Y, Shi X, Liu T, Chen Z, Zuo X (2021). Circular RNA 100146 promotes colorectal cancer progression by the MicroRNA 149/HMGA2 Axis. Mol Cell Biol.

[31] Li L, Wei H, Zhang H, Xu F, Che G (2020). Circ_100565 promotes proliferation, migration and invasion in non-small cell lung cancer through upregulating HMGA2 via sponging miR-506-3p. Cancer Cell Int.

[32] Cheng Y, Huang C, Mo Y, Wu W, Liang L. WITHDRAWN: long non-coding RNA UCA1 regulates tumor growth by impairing let-7e-dependent HMGA2 repression in bladder cancer. Cancer Biomark. 2019. 10.3233/CBM-182296. 10.3233/CBM-182296 31306103

[33] Lee J, Ha S, Jung CK, Lee HH (2015). High-mobility-group A2 overexpression provokes a poor prognosis of gastric cancer through the epithelial-mesenchymal transition. Int J Oncol.

[34] Ye J, Liu J, Tang T, Xin L, Bao X, Yan Y (2021). LINC00963 affects the development of colorectal cancer via MiR-532-3p/HMGA2 axis. Cancer Cell Int.

