# Supplementary material for: Circ_0000658 knockdown inhibits epithelial-mesenchymal transition in bladder cancer via miR-498-induced HMGA2 downregulation
Source: J Exp Clin Cancer Res. 2022 Jan 14;41:22. doi: 10.1186/s13046-021-02175-3 (PMC8759287; doi:10.1186/s13046-021-02175-3)
Supplement: Supplementary file 4 — Additional file 4: Table S1. Clinicopathological characteristics of 50 patients with BCa. Table S2. Primer sequences used for RT-qPCR. Table S3. MiRNAs downstream circ_0000658 based on RegRNA 2.0 and circInteractome databases. Table S4. Intersection of downstream target genes of miR-498 predicted by StarBase and miRDB databases with the BCa-related genes in the GeneCards database. [file 13046_2021_2175_MOESM4_ESM.docx]

**Table S1** Clinicopathological characteristics of 50 patients with BCa

| Variables | n | circ_0000658 expression | | *p* value |
| --- | --- | --- | --- | --- |
|  |  | high | low |  |
| Sex |  |  |  | 0.4962 |
| Male | 39 | 18 | 21 |  |
| Female | 11 | 7 | 4 |  |
| Age (year) |  |  |  | 0.4635 |
| < 50 | 9 | 6 | 3 |  |
| ≥ 50 | 41 | 19 | 22 |  |
| T stage |  |  |  | 0.0003 |
| T1+T2 | 36 | 12 | 24 |  |
| T3+T4 | 14 | 13 | 1 |  |
| N stage |  |  |  | 0.0005 |
| N0 | 27 | 7 | 20 |  |
| N1 | 23 | 18 | 5 |  |
| TNM |  |  |  | 0.0011 |
| Ⅰ | 16 | 4 | 12 |  |
| Ⅱ | 11 | 3 | 8 |  |
| Ⅲ | 23 | 18 | 5 |  |

**Table S2** Primer sequences used for RT-qPCR.

| Gene | Primer sequence |
| --- | --- |
| circ_0000658 | Forward: 5'-GGCCTCGCACAGATACCC-3' |
|  | Reverse: 5'-ATGGCTGCATGAAGACCCAA-3' |
| GAPDH | Forward: 5'-GGAGCGAGATCCCTCCAAAAT-3' |
|  | Reverse: 5'-GGCTGTTGTCATACTTCTCATGG-3' |
| miR-498 | Forward: 5’-TTTCAAGCCAGGGGGCGTTTTTC-3’， |
|  | Reverse: 5’-CGACAGTTGCTATGCGATGCA-3’ |
| HMGA2 | Forward: 5′-ACCCAGGGGAAGACCCAAA-3′ |
|  | Reverse: 5′-CCTCTTGGCCGTTTTTCTCCA-3′ |
| E-cadherin | Forward: 5'-CGAGAGCTACACGTTCACGG-3' |
|  | Reverse: 5'-GGGTGTCGAGGGAAAAATAGG-3' |
| Slug | Forward: 5′-CGAACTGGACACACATACAGTG-3′ |
|  | Reverse: 5′-CTGAGGATCTCTGGTTGTGGT-3′ |
| Snail | Forward: 5′-TCGGAAGCCTAACTACAGCGA-3′ |
|  | Reverse: 5′-AGATGAGCATTGGCAGCGAG-3′ |
| ZEB1 | Forward: 5′-GATGATGAATGCGAGTCAGATGC-3′ |
|  | Reverse: 5′-ACAGCAGTGTCTTGTTGTTGT-3′ |
| Twist | Forward: 5'-GTCCGCAGTCTTACGAGGAG-3' |
|  | Reverse: 5'-GCTTGAGGGTCTGAATCTTGCT-3' |
| N-cadherin | Forward: 5'-TTTGATGGAGGTCTCCTAACACC-3' |
|  | Reverse: 5'-ACGTTTAACACGTTGGAAATGTG-3' |
| U6 | Forward: 5'-AAAGCAAATCATCGGACGACC-3 ' |
|  | Reverse: 5'-GTACAACACATTGTTTCCTCGGA-3' |

**Table S3** MiRNAs downstream circ_0000658 based on RegRNA 2.0 and circInteractome databases

CircInter|RegRNA 2.0

hsa-miR-1200

hsa-miR-1229

hsa-miR-1236

hsa-miR-1248

hsa-miR-1287

hsa-miR-1303

hsa-miR-140-3p

hsa-miR-146b-3p

hsa-miR-498

hsa-miR-512-5p

hsa-miR-548b-3p

hsa-miR-586

hsa-miR-591

hsa-miR-610

hsa-miR-637

hsa-miR-640

hsa-miR-663b

hsa-miR-671-5p

hsa-miR-758

hsa-miR-767-5p

hsa-miR-769-5p

hsa-miR-874

hsa-miR-892a

hsa-miR-936

hsa-miR-938

hsa-miR-942

**Table S4** Intersection of downstream target genes of miR-498 predicted by StarBase and miRDB databases with the BCa-related genes in the GeneCards database.

StarBase|miRDB|GeneCards

KLF6

BMI1

WT1

HMGA2

DICER1

CREBBP

WWOX

ODC1

ZEB2

CREB1

EP300

PIK3R1

GJA1

CDK6

TSC1
